# Overexpression of AtGRDP2, a novel glycine-rich domain protein, accelerates plant growth and improves stress tolerance

**DOI:** 10.3389/fpls.2014.00782

**Published:** 2015-01-20

**Authors:** María A. Ortega-Amaro, Aída A. Rodríguez-Hernández, Margarita Rodríguez-Kessler, Eloísa Hernández-Lucero, Sergio Rosales-Mendoza, Alejandro Ibáñez-Salazar, Pablo Delgado-Sánchez, Juan F. Jiménez-Bremont

**Affiliations:** ^1^División de Biología Molecular, Instituto Potosino de Investigación Científica y Tecnológica ACSan Luis Potosí, México; ^2^Facultad de Ciencias, Universidad Autónoma de San Luis PotosíSan Luis Potosi, Mexico; ^3^Facultad de Ciencias Químicas, Universidad Autónoma de San Luis PotosíSan Luis Potosi, Mexico; ^4^Facultad de Agronomía, Universidad Autónoma de San Luis PotosíSan Luis Potosi, México

**Keywords:** glycine-rich domain protein, *Arabidopsis thaliana*, *Lactuca sativa*, development, indole-3-acetic acid, salt stress

## Abstract

Proteins with glycine-rich signatures have been reported in a wide variety of organisms including plants, mammalians, fungi, and bacteria. Plant glycine-rich protein genes exhibit developmental and tissue-specific expression patterns. Herein, we present the characterization of the *AtGRDP2* gene using Arabidopsis null and knockdown mutants and, Arabidopsis and lettuce over-expression lines. *AtGRDP2* encodes a short glycine-rich domain protein, containing a DUF1399 domain and a putative RNA recognition motif (RRM). *AtGRDP2* transcript is mainly expressed in Arabidopsis floral organs, and its deregulation in Arabidopsis *Atgrdp2* mutants and *35S::AtGRDP2* over-expression lines produces alterations in development. The *35S::AtGRDP2* over-expression lines grow faster than the WT, while the *Atgrdp2* mutants have a delay in growth and development. The over-expression lines accumulate higher levels of indole-3-acetic acid and, have alterations in the expression pattern of *ARF6*, *ARF8*, and *miR167* regulators of floral development and auxin signaling. Under salt stress conditions, *35S::AtGRDP2* over-expression lines displayed higher tolerance and increased expression of stress marker genes. Likewise, transgenic lettuce plants over-expressing the *AtGRDP2* gene manifest increased growth rate and early flowering time. Our data reveal an important role for *AtGRDP2* in Arabidopsis development and stress response, and suggest a connection between *AtGRDP2* and auxin signaling.

## Introduction

Glycine-rich proteins (GRPs) are characterized by a high content of glycine (40–70%) and repetitive sequence of residues arranged in (Gly)_n_-X motifs (Sachetto-Martins et al., 2000; Mousavi and Hotta, 2005). Proteins with particular glycine-rich regions have been reported in a wide variety of organisms including plants (Sachetto-Martins et al., 2000). Plant *GRP* genes exhibit developmentally regulated and tissue-specific expression patterns; these patterns are also regulated by abiotic and biotic factors (Sachetto-Martins et al., 2000).

The plant GRP family is classified according to its general structure. Currently, five groups have been suggested, according to the arrangement of the repeated glycine signatures as well as the presence of conserved motifs and domains (Sachetto-Martins et al., 2000; Mangeon et al., 2010). The first three classes and the fifth are based on the arrangement of the glycine-rich domain, i.e., (I) GGX, (II) GGXXXGG, (III) GXGX, and (V) GGX/GXGX, respectively; they also contain a signal peptide, oleosin, or cysteine-rich domain. The group IV is based on the presence of additional motifs and domains such as RNA recognition motif (RRM), cold shock and zinc finger domains, and a cysteine-rich domain, among others (Bocca et al., 2005; Mangeon et al., 2010). Until now, few plant GRPs have been characterized; these proteins appear to play important roles in transcriptional regulation, signal transduction, protein-protein interaction, development, and stress responses (Bocca et al., 2005).

GRPs are involved in developmental processes in plants. The bean PvGRP1.8 has been proposed to act as an agglutinating agent for deposition of cell wall constituents (Keller et al., 1988) and it has been associated with protoxylem growth (Ryser et al., 1997). In stems and leaves of Petunia plants, the levels of *PtGRP1* gene decline with the developmental age of the tissue. In particular, *PtGRP1* gene expression levels were associated to expansive growth, and the PtGRP1 protein was localized in the cell wall/membrane interphase (Condit, 1993). In the past years, it has been documented that some plant hormones that regulate many aspects of plant growth, development and stress responses modulate the expression of GRP genes (Reddy and Poovaiah, 1987; Urbez et al., 2006; Long et al., 2013).

In addition to the canonical GRPs, Bocca et al. (2005) reported the presence of glycine-rich domain proteins (GRDPs) transcripts in *Eucalyptus*, which encode proteins with a short glycine-rich domain. Recently, we reported the *AtGRDP1* gene, encoding a short glycine-rich domain protein, which plays a regulatory role in ABA signaling and abiotic stress tolerance (Rodríguez-Hernández et al., 2014).

To deepen our understanding in GRDPs, herein we present the characterization of *AtGRDP2* gene, paralog of *AtGRDP1*. Arabidopsis plants that overexpress the *AtGRDP2* gene were obtained. These plants grow faster and flower earlier than the WT, while *Atgrdp2* knockout and knockdown mutants have a delay in growth and development. The over-expression of *AtGRDP2* in lettuce plants also conduces to increased growth rate and early flowering time. In Arabidopsis, *AtGRDP2* gene is highly expressed in floral organs, and is auxin-responsive. We further show that *35S::AtGRDP2* overexpression lines accumulate higher levels of indole-3-acetic acid, which might explain their fast growth. We analyzed central regulators in auxin signaling such as *ARF2*, *ARF6, ARF8*, *AUX1*, and *miR167*, and found that these genes are differentially expressed in *Atgrdp2*-*1* mutant and *35S::AtGRDP2* overexpression lines. Under salt stress conditions, seedlings of *35S::AtGRDP2* lines displayed higher tolerance and increased expression of stress marker genes. Our data reveal an important role for *AtGRDP2* in Arabidopsis development and stress responses, possibly through an auxin-dependent mechanism.

## Materials and methods

### Plant material and growth conditions

The mutant and transgenic lines used in this study were generated in the *Arabidopsis thaliana* ecotype Columbia 0 (Col-0) background. Arabidopsis seeds of each line were surface-sterilized for 10 min with 40% (v/v) chlorine solution and rinsed six times in sterile distilled water. Aseptic stratified seeds (2 days at 4°C), were germinated and grown on agar plates containing 0.5x Murashige and Skoog (MS) medium, pH 5.7, 0.5% (w/v) sucrose, and 1.2% (w/v) agar (Murashige and Skoog, 1962). Plates were incubated in a growth chamber with a photoperiod of 16 h (13,000 luxes) / 8 h, light/dark cycle at a temperature of 22 ± 2°C. Plants were grown in plastic pots with a mixture of Sunshine Mix#3 commercial substrate and vermiculite (3:1), under environmental controlled conditions.

Seeds of *Lactuca sativa* L. (WT) and seeds of *35S::AtGRDP2* transgenic lettuce plants were sterilized with 20% (v/v) chlorine solution for 10 min, and rinsed five times in sterile distilled water. Aseptic seeds were germinated in Petri dishes containing 0.5x MS medium. Plates were kept at 4°C for 2 days and then incubated at 22 ± 2°C for 7 days in a growth chamber under a 16 h light/8 h dark photoperiod. Afterwards, plants were transferred to soil pots in a growth chamber at 25 ± 2°C with a 16 h light/8 h dark photoperiod.

### Identification of the T-DNA insertional mutant line (*Atgrdp2-1*)

The T-DNA mutant line Sail_387_D04 for the *AtGRDP2* gene (At4g37900) was acquired from the Salk Institute Genome Analysis Laboratory (www.signal.salk.edu/cgi-bin/tdnaexpress; Alonso et al., 2003). Absence of *AtGRDP2* expression in T-DNA mutant line was confirmed by semi-quantitative RT-PCR using the Sail387D04-F and Sail387D04-R primers (Table S1). cDNA synthesis was carried out using 1 μg of total RNA and the SuperScript™ First-Strand Synthesis System (Invitrogen). The *Actin 8* gene (At1g49240) was amplified as loading control using the ACT8-F and ACT8-R primers (Table S1).

### Vectors for *AtGRDP2* overexpression and gene silencing

*AtGRDP2* ORF was amplified from cDNA of 15 day-old Arabidopsis plants with Hot Star HiFidelity Polymerase Kit (Qiagen, Hilden, Germany) using the primers: AtGRDP2-ORF-F and AtGRDP2-ORF-R primers (Table S1). The product of 2377 bp was cloned into the pCR8/GW/TOPO vector (Invitrogen, Carlsbad, CA, USA), and was sequenced using the M13-F and AtGRDP2-ORF-R primers. The entry clone was recombined into the destination vector pMDC32 using the Gateway LR Clonase Enzyme mix (Invitrogen) to generate *pMDC32-GRDP2* vector.

To silence the *AtGRDP2* gene, an artificial miRNA *pAmiR-AtGRDP2* vector from Thermo Fisher Scientific Inc. was acquired (Waltham, MA, USA). This vector contains 27 bp of the *AtGRDP2* gene between the *miR319a* harpin sequence embedded in their genomic context, *35S* CaMV promoter, and the BASTA resistance (Schwab et al., 2006).

### Transformation of Arabidopsis and lettuce

The vectors *pMDC32-GRDP2* and *pAmiR-AtGRDP2* were transferred into *Agrobacterium tumefaciens* GV2260 strain by electroporation, and transformed into *A. thaliana* Col-0 plants by the floral dip method (Clough and Bent, 1998). Afterwards, seeds were harvested for selection under a specific plant selection marker. Hygromycin was used at 50 μg/mL for *pMDC32-GRDP2* selection. Ten independent *35S::AtGRDP2* lines were obtained, and four T3 homozygous lines were used for further analysis. For *pAmiR-AtGRDP2* selection, 1-week-old germinated seedlings were sprayed using a 0.25 g/L BASTA (Finale®, Bayer Cropscience, Monheim, Germany). Five independent *amiR-AtGRDP2* T3 homozygous lines were obtained.

Lettuce plants carrying the *AtGRDP2* gene were generated by *Agrobacterium*-mediated transformation, following Curtis et al. (1994) method with some modifications (Martinez-Gonzalez et al., 2011). Nine lettuce transgenic lines were obtained in F1 generation, and the presence of the transgene was confirmed by PCR. T3 homozygous seedlings of three transgenic plants were used for further analysis.

### Quantitative RT-PCR (qRT-PCR) of *AtGRDP2* gene in Arabidopsis Col-0 plants

Total RNA was obtained from different tissues of 18, 21, 26, 28, and 45 days old *A. thaliana* Col-0 plants. *AtGRDP2* expression levels were estimated by qRT-PCR as described below using the following primers: AtGRDP2-F and AtGRDP2-R (Table S1). For each tissue, three biological replicates were analyzed with their respective technical replicates. Each biological replicate consisted in groups of 10 seedlings.

### Expression analysis of mutant and overexpression lines by qRT-PCR

Arabidopsis RNA extractions were done with 100–200 mg of plant material following the Concert™ Plant RNA Reagent protocol (Invitrogen). Each mRNA biological replicate consisted in groups of 10 plants per line. Contamination of genomic DNA was eliminated by treatment with DNase I (Invitrogen). *AtGRDP2* expression levels in Arabidopsis *amiR* and *35S::AtGRDP2* overexpression lines was estimated from 15 days old plants, using the primers: AtGRDP2-F and AtGRDP2-R (Table S1). cDNA synthesis and quantitative PCR analyses were done in a 10 μL reaction mixture containing 50 ng of total RNA as template using the Power SYBR® Green RNA-to-CT™ One-Step Kit (Applied Biosystems). The thermal cycling conditions consisted of 30 min at 48°C (cDNA synthesis), 10 min at 95°C (activation of AmpliTaq Gold® DNA polymerase), followed by 40 PCR cycles of 15 s at 95°C (denature) and 1 min at 60°C (anneal/extend). Melting curves were performed by cycles of 15 s at 95°C (denature), 15 at 60°C (anneal) and 15 s at 95°C (denature), increasing the temperature each 0.3°C. The cycle number at threshold (Ct value) was used for calculations of relative mRNA expression levels. The Ct value of each target gene was normalized by subtraction of the Ct value from the Arabidopsis *ubiquitin 5* (*At3g62250*) gene. The fold change in gene expression relative to control samples (Col-0) was calculated using the 2^−ΔΔCt^ method (Livak and Schmittgen, 2001). For each sample, three biological replicates were analyzed with their respective technical replicates.

Lettuce RNA extractions were done with 100–200 mg of plant material following the Concert™ Plant RNA Reagent protocol (Invitrogen). Contamination of genomic DNA was eliminated by treatment with DNase I (Invitrogen). *AtGRDP2* expression levels in transgenic lettuce were also estimated from 15 days old plants, using the AtGRDP2-F and AtGRDP2-R primers (Table S1). Relative gene expression levels of *AtGRDP2* gene in transgenic lettuce are presented as 2^−ΔCt^, where ΔCt = Ct_*AtGRDP2*_ − Ct*_LsUBQ5_*. For the amplification of the lettuce *LsUBQ5* gene, the LsUBQ5-F and LsUBQ5-Rv primers were used (Table S1). For each sample, three biological replicates were analyzed with their respective technical replicates.

### Analysis of *AtGRDP2* promoter::GUS-GFP expression

The *AtGRDP2* promoter region (2 kb upstream of the start codon) was PCR-amplified from the Arabidopsis genomic DNA. Amplification was carried out with primers ATPROM37fw and ATPROM37rv (Table S1). The fragment was cloned into pCR®8/GW/TOPO® entry vector (Invitrogen) and fused by recombination to the GUS-GFP reporter genes in the pKGWFS7 binary vector (Karimi et al., 2002). *Agrobacterium tumefaciens* GV2260 strain harboring the *AtGRDP2* promoter::GUS-GFP construction was used for Arabidopsis transformation, as described before. Five independent transgenic lines were selected on 50 μg/mL kanamycin. T3 homozygous seedlings were used for GUS histochemical analysis as described below.

### Histochemical analysis in Arabidopsis *pAtGRDP2*::*GUS-GFP* reporter lines

Expression patterns of the 45 day-old Arabidopsis *pAtGRDP2::GUS-GFP* reporter lines were analyzed. For GUS staining, two independent lines (*pAtGRDP2::GUS-GFP*-1 and *pAtGRDP2::GUS-GFP*-2) were used, following the protocol described previously (Ortega-Amaro et al., 2012). For each marker line, 10 transgenic plants were analyzed. A representative plant was chosen and photographed using MOTIC model BA-300 microscope and processed with MOTIC software (version 2.0) using a 5.0 megapixels camera. The anatomy of Arabidopsis flower was described according to Roeder and Yanofsky (2006). All experiments were repeated at least twice obtaining similar results.

### Measurement of flowering time

Flowering time in Arabidopsis in both short (8 h/16 h light/dark cycle) and long (16 h/8 h light/dark cycle) day conditions was estimated by recording the number of days after sowing in which the inflorescence reached 1 cm in length; in addition, at the same time the rosette leaves were counted. Flowering time in lettuce plants was recorded daily as of the appearance of the floral bud, and data were graphically represented as the percentage of plants with floral tissues.

### Histochemical analysis in Arabidopsis *pAtGRDP2::GFP-GUS* lines under IAA treatments

Seven day-old *pAtGRDP2::GFP-GUS-1* and *pAtGRDP2::GFP-GUS-2* seedlings grown in 0.5x MS plates were transferred to 0.5x MS liquid medium supplemented with 0, 1, and 10 μM IAA and, incubated for 3 and 6 h in a grown chamber under controlled conditions.

For each marker line and for each treatment, 10 transgenic plants with three replicates were analyzed. After each treatment, seedlings were subjected to GUS histochemical analysis as described before. Images of *pAtGRDP2::GFP-GUS-1* and *pAtGRDP2::GFP-GUS-2* lines were recorded. A representative plant was chosen and photographed using Leica microscope at 10X magnification and recorded using the Leica Application Suite 3.0.0 software.

### IAA content on Arabidopsis by elisa assay

For IAA estimation 30 day-old *A. thaliana* Col-0*, Atgrdp2-1*, and *amiR-1* mutants and *AtGRDP2* over-expression lines were used. The extract was obtained from 1 g of ground plant tissue, and incubated overnight in 80% methanol at 4°C. Methyl groups were added by the addition of 1.3 μl trimethylsilyldiazomethane. Next, the samples and the IAA standard were processed by the manufacturer's protocol (Phytodetek® IAA Test Kit, Agdia, USA). Absorbance values at 405 nm were obtained using a BioTek ELx800 microplate reader, and then the concentration of IAA was calculated based on standard curve. Standard curve and IAA estimation in Arabidopsis extracts (1:20 dilution) were performed on three biological replicates with their respective technical replicates.

### Expression of auxin related genes in Arabidopsis

The expression level of auxin related genes in the Col-0, *Atgrdp2-1*, and *35S::AtGRDP2-OE3* overexpression lines was analyzed by qRT-PCR. The auxin related genes *ARF6* (At1g30330), *ARF8* (At5g37020), *ARF2* (At5g62000), *AUX1* (At2g38120) were measured in 21 days old plants. For the estimation of *miR167* (At3g04765) expression levels, 1 μg of total RNA was used for small RNA polyadenylation process and cDNA production following the NCode miRNA cDNA synthesis kit (Invitrogen) manufacture directions. qPCR was performed with SYBR Green qPCR Master Mix (Thermo scientific) protocol. Quantitation was based on a cycle threshold value (Livak and Schmittgen, 2001). Specific primers for each gene are indicated in table S1. For each sample, three biological replicates were analyzed with their respective technical replicates. Each biological replicate consisted in groups of 10 seedlings. In case of ratios lower than 1, the inverse of the ratio was estimated and the sign was changed.

### Measurement of main root size and fresh weight of seedlings

*A. thaliana* seeds of the Col-0, the *Atgrdp2*-*1* and *amiR-1* mutants, and the *35S::AtGRDP2* overexpression lines were grown on 0.5x MS plates for 14 days. Estimation of main root size (cm) was determined by measuring the length of 30 plant roots of each line. Fresh weight (mg) of seedlings was obtained on an analytical scale and the values obtained represent means of three groups of 10 seedlings for line. Estimation of lettuce (WT and transgenic plants) main root size (cm), stem length (cm) and fresh weight (mg) was obtained from 9 seedlings of each line. Statistical significance among data was estimated by One-way-ANOVA and Tukey's multiple comparison post-test, using GraphPad Prism version 5.0 (GraphPad Software, California, USA). The experiment was repeated three times with similar results.

### Arabidopsis salinity stress assay *in vitro*

Fourteen days-old Arabidopsis Col-0, *Atgrdp2-1* and overexpression lines (*35S::AtGRDP2*) grown on MS 0.5x, were transferred into 0.5x MS plates supplemented with 0 or 150 mM NaCl. After 7 days of stress, the main root size of untreated and treated plantlets was estimated measuring 30 roots of each line. The relative root length was calculated (treated with 150 mM NaCl/untreated). Fresh weight (mg) of seedlings was obtained on an analytical scale and the values obtained represent means of three groups of 10 seedlings of each line. The relative fresh weight was calculated (treated with 150 mM NaCl /untreated). After 7 days of salt stress, groups of 10 seedlings of each line were transferred to three pots with soil mixture substrate, grown under controlled conditions (22 ± 2°C; 16 h light/8 h dark photoperiod) and irrigated every 3 days, to observe post-stress recovery. Plant survival rate was calculated by counting the number of plants that survived in each pot, after a period of 14 days. The experiment was repeated three times with similar results.

### Lettuce salinity stress assay *in vitro*

Lettuce seeds of WT and transgenic plants were germinated on MS medium supplemented with 0 and 150 mM NaCl. After 21 days of treatment, data of root length and fresh weight were recorded. Estimation of lettuce (WT and transgenic plants) main root length and fresh weight was obtained from 9 seedlings of each line. The relative root length was calculated (treated with 150 mM NaCl/untreated). These stress experiments were repeated three times obtaining similar results.

### Expression of salt stress related genes in Arabidopsis

Fourteen days old Col-0, *Atgrdp2-1* and *35S::AtGRDP2-OE3* overexpression line, grown on 0.5x MS plates were used. Ten seedlings of each line in triplicate manner were transferred to 0.5x MS medium with 0, 125, and 150 mM NaCl and, incubated for 1 and 3 days in a growth chamber under controlled conditions. After, plantlets of each treatment were frozen in liquid nitrogen and total RNA extraction was performed as described before. RNA was used for expression analysis of *RD29B* (At5g52300), *EM6* (At2g40170), and *ABF4* (At3g1929) genes by qRT-PCR. Specific primers are indicated in Table S1. For each sample, three biological replicates were analyzed with their respective technical replicates. Each biological replicate consisted of groups of 10 seedlings. In case of ratios lower than 1, the inverse of the ratio was estimated and the sign was changed.

### Measurement of chlorophyll and carotenoids content in lettuce plants

The chlorophylls *a*, *b*, total chlorophyll, and carotenoids contents were obtained from 30, 60, and 90 days old WT and transgenic lettuce plants. Three samples (leaf disc) of four plants of each line were collected. The pigments were extracted according to the procedure of Hendry and Price (1993). Samples of 50 mg fresh weight were macerated with 1 mL 80% acetone. These samples were centrifuged at 3000 g for 2 min and the absorbance of each supernatant was determined with the following wavelengths: 645 and 663 nm for chlorophylls *a* and *b*, and at 470 nm for total carotenoids, using a spectrophotometer (Thermo Scientific®, Model Spectronic Genesys 10 Bio).

### Sequence analysis

Comparisons and protein sequence alignments were carried out using the CLUSTAL W and the T-Coffee programs at the EBI database (www.ebi.ac.uk). The amino acid sequences of the AtGRDP2 (At4g37900) protein and selected plant orthologous and *A. thaliana* paralogous proteins containing the DUF1399 domain were aligned using the T-Coffee Multiple Sequence Alignment Tool of the EBI database using default values (Notredame et al., 2000). The aligned sequences were subjected to re-sampling with replacement (1000 bootstrap) using the Seqboot program and subsequently analyzed by a distance method in the PHYLIP 3.67 package (Felsenstein, 1989). The distance matrix was calculated with the Protdist program using the Henikoff/Tillier Probability Matrix from Blocks (PMB, Veerassamy et al., 2003). The resultant matrices were then transformed into multiple trees by the Neighbor Joining program and summarized by the program Consense (both programs of the PHYLIP 3.67 package) (Felsenstein, 1989). The majority rule consensus tree was edited with the MEGA version 5.0 program (Tamura et al., 2011).

## Results

### Arabidopsis *AtGRDP2* gene encodes a glycine-rich domain protein

The *AtGRDP2* (*Arabidopsis thaliana* glycine-rich domain protein 2) gene encodes a glycine-rich domain protein. The *AtGRDP2* (At4g37900) gene is located in chromosome 4, and its genomic organization consists of five exons and four introns. The *AtGRDP2* cDNA is 2428 bp in length containing an open reading frame (ORF) of 2364 pb that encodes a protein of 787 aa. Protein sequence analyses revealed a domain of unknown function (DUF1399), a putative RNA binding motif (RNP), and a glycine-rich domain (GRD) in the AtGRDP2 protein (Figure S1A). The DUF1399 domain is located in the N-terminus and consists of 142 aa. The putative RNA binding motif (KGSCFLPM) is placed in the central region of the protein, and it is equivalent to RNP-1 (ribonucleoprotein-1) present in proteins with RNA chaperone activity (Lorković and Barta, 2002). The glycine-rich domain is found in the C-terminus (aa 713-766); this domain also contains interspersed cysteine residues (Figure S1A). Comparison of phylogenetically related GRDPs reveals that the three domains found in AtGRDP2 are conserved in the orthologous proteins analyzed (Figures S1, S2). AtGRDP2 is grouped with orthologous GRDPs of dicot plants (Figure S1B), mainly with proteins from the *Brassicaceae* species. A consensus glycine region [CG]GGGCGG[GC], elucidated by MEME program (Bailey and Elkan, 1994), was identified among the AtGRDP2 orthologs (Figures S1A, S2).

In the *A. thaliana* genome, in addition to AtGRDP2, we have identified three additional genes encoding proteins that also contain the DUF1399 domain, named AtGRDP1 (Rodríguez-Hernández et al., 2014), At1g56230, and At4g37682. The AtGRDP1 protein also contains the RNP-1 and glycine rich-region domains, while the At1g56230 and At4g37682 encoded proteins lack these domains (Figure S1C).

### *AtGRDP2* is expressed throughout Arabidopsis development

**Figure 1:**
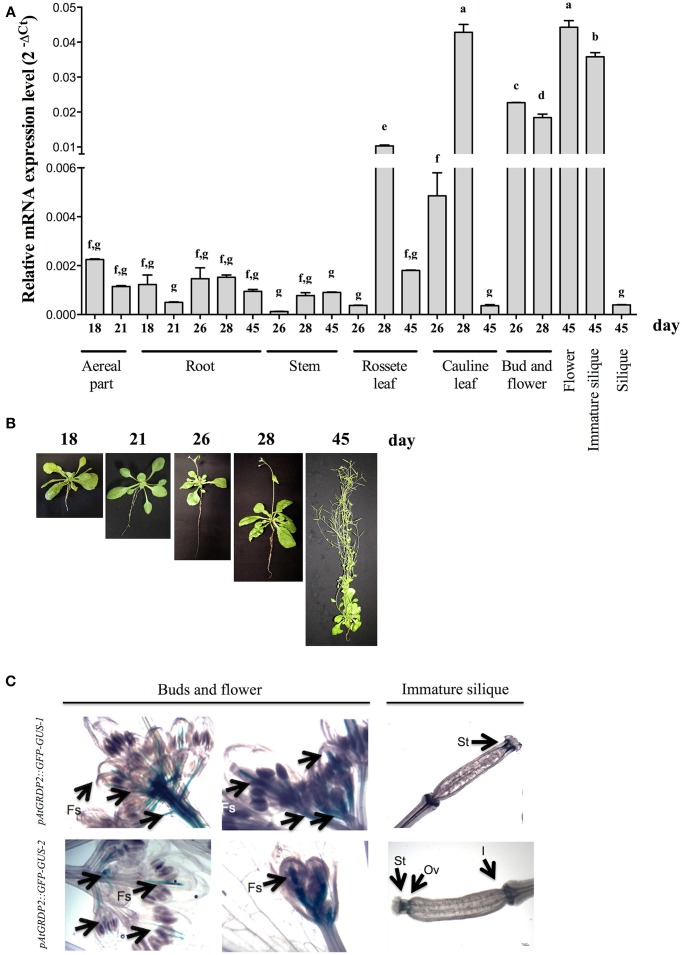
***AtGRDP2* is expressed throughout Arabidopsis development. (A)** qRT-PCR analysis was performed in Arabidopsis Col-0 tissues obtained from different developmental stages. Quantitation of the *AtGRDP2* gene, expressed as relative mRNA expression levels (2^−ΔCt^), was calculated after normalization to the Arabidopsis *UBQ5* gene. For each sample, three biological replicates were analyzed with their respective technical replicates. Letters indicate significant differences between samples according to Tukey's multiple comparison tests at *P* < 0.05. **(B)** Photographs show representative Arabidopsis plant at 18, 21, 26, 28 and 45 days. **(C)** Representative images of GUS histochemical staining of 45 day-old transgenic lines *AtGRDP2::GFP-GUS*-1 and -2 in buds, flowers and immature siliques; fertilized siliques (Fs), stigmas (St), internode region (I), and ovary (Ov) are indicated.

*AtGRDP2* tissue-specific expression pattern was evaluated by qRT-PCR at different developmental stages, in *A. thaliana* ecotype Col-0 plants of 18, 21, 26, 28, and 45 days old (Figures 1A,B). The highest *AtGRDP2* expression levels were found in rosette leaves, in inflorescence tissues such as cauline leaves, buds, flowers, and in immature siliques (Figure 1A). In rosette and cauline leaves, the highest expression was detected in 28-day-old plants. In flowers, the maximum expression of *AtGRDP2* was noticed at day 45; although expression in buds and flowers was detected as of day 26. Furthermore, *AtGRDP2* gene expression was maintained in immature siliques, but expression fell toward baseline values in mature siliques (Figure 1A).

*AtGRDP2* expression in floral tissues was also evaluated using Arabidopsis reporter lines expressing the GFP-GUS fusion under the control of the *AtGRDP2* promoter (2000 bp) (Figure 1C). GUS histochemical assays confirmed *AtGRDP2* expression in flower organs and immature siliques (Figure 1C). GUS expression was detected on the stigmas (St), at the top of the ovary (Ov), internode region (I), and in floral buds and fertilized siliques (Fs).

### *AtGRDP2* gene is involved in Arabidopsis development

To address the biological functions of *AtGRDP2* gene in plant growth and development, mutant and over-expression lines were characterized. The T-DNA homozygous line (Sail_387D04) was analyzed for the absence of *AtGRDP2* transcript, confirming that the Sail_387D04 line is a null allele (Figure S3). The *amiRAtGRDP2-1* line (−1.7-fold repression), and four Arabidopsis lines over-expressing the *AtGRDP2* gene (*35S::AtGRDP2-OE2* 4.8-fold, *-OE4* 21.6-fold, *-OE1* 102.6-fold, and *-OE3* 451.3-fold) were selected for subsequent analyses (Figure S3D).

**Figure 2:**
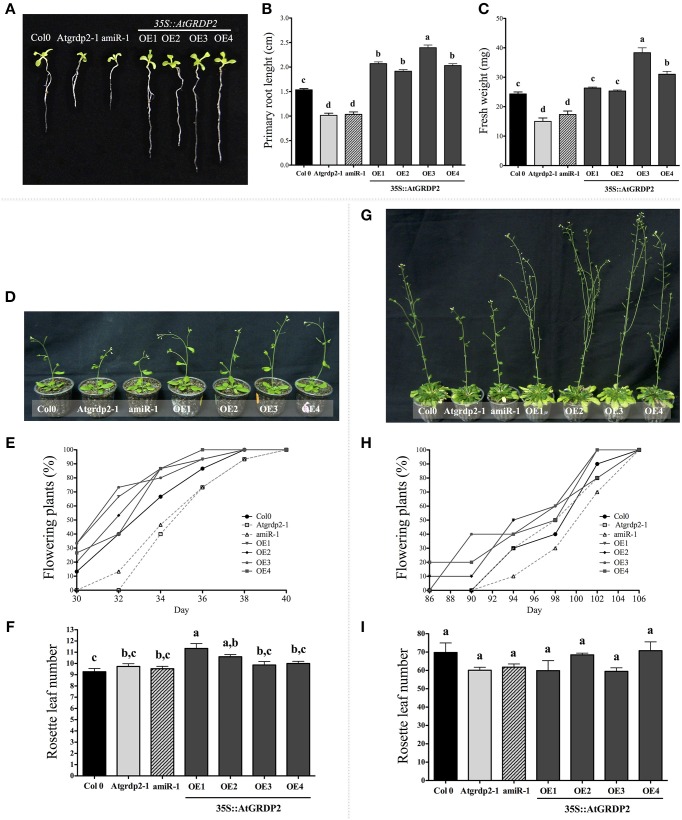
**Growth of Col-0, *Atgrdp2*-*1*, *amiR-1*, and *AtGRDP2* overexpression lines. (A)** Phenotype of 14-day-old WT, mutants and overexpression seedlings. Primary root length **(B)** and fresh weight **(C)** were measured. Estimation of main root length (cm) was determined by measuring the length of 30 plant roots of each line. The fresh weight (mg) of the seedlings was obtained on an analytical scale and the values obtained represent the means of three groups of 10 seedlings of each line. Estimation of flowering time in plants grown under long days. **(D)** Phenotype of 40 day-old plants, **(E)** distribution of flowering plants, and **(F)** rosette leaf number in Col-0, mutant, and over-expression lines (mean ± SE *n* = 15). Estimation of flowering time in plants grown under short days. **(G)** Phenotype of 110 day-old plants, **(H)** distribution of flowering plants and **(I)** rosette leaf number in Col-0, mutant and over-expressing lines (mean ± SE *n* = 15). Error bars denote SE and significant differences are indicated with different letters. One-Way ANOVA was used to analyze the data (*P* < 0.05) and differences among treatments were explored through Tukey's multiple comparisons tests.

Growth rate was evaluated in 2-week-old Arabidopsis *Atgrdp2* mutants and *AtGRDP2* over-expression lines. At this stage, seedlings of knockout and knockdown lines exhibited smaller sizes than those of WT plantlets (Figure 2A), reflected in their root length and fresh weight (Figures 2B,C). Interestingly, the *AtGRDP2* over-expression lines presented an opposite phenotype, resulting in a higher growth rate in comparison to WT seedlings, in both the aerial part and the roots (Figure 2A). The increased growth rate observed in *35S::AtGRDP2-OE3* plantlets could be attributed to a higher accumulation of *AtGRDP2* transcript in this line (Figure S3). Since deregulation of *AtGRDP2* gene results in plant growth alterations, we analyzed the flowering time under long- and short-day conditions (Figures 2D–I). In long-day conditions, *AtGRDP2* over-expression lines showed faster development and early flowering in comparison with the WT (Figures 2D,E). Instead, the knockout and knockdown lines showed late flowering phenotype (Figures 2D,E). At day 34, the percentages of plants with inflorescences were as follows: >80% in the *AtGRDP2* over-expression lines, 66% in the WT, 46.6% in the knockdown line and 40% in the knockout line. Regarding the number of rosette leaves, no significant differences were observed in most of the analyzed lines. The exception was in the *35S::AtGRDP2-OE1* which had more rosette leaves (Figure 2F).

Flowering time experiments under short-day conditions exhibited similar behavior to that observed in plants grown in long-days. The *AtGRDP2* over-expression lines showed early flowering in comparison with WT plants, knockout and knockdown lines (Figures 2G–I). Finally, no differences in the number of rosette leaves at the flowering time were found between WT and the analyzed lines (Figure 2I).

**Figure 3:**
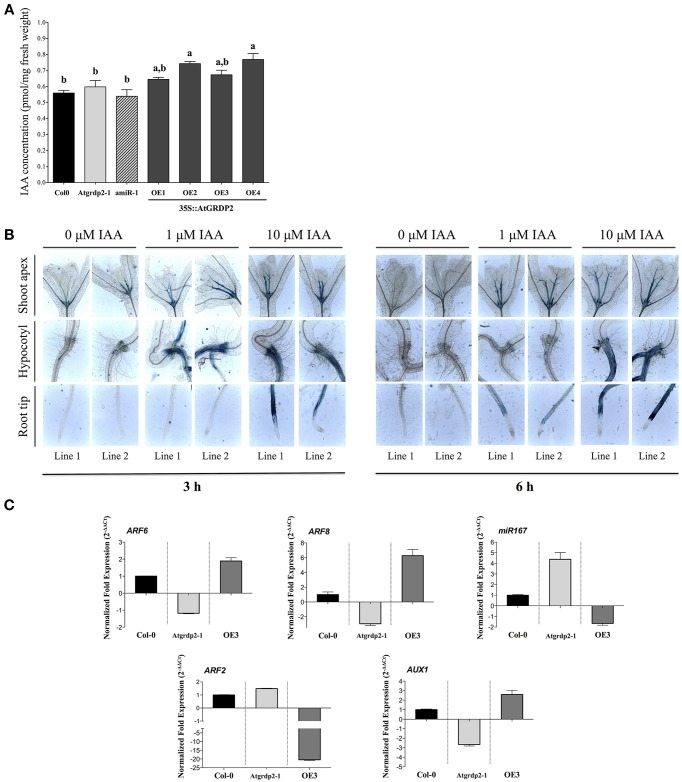
**(A)** IAA content in Col-0, *Atgrdp2-1*, *amiR-1*, and *AtGRDP2-OE1, OE2, OE3*, and *OE4* overexpression lines. Twenty-one days old plants were used for IAA quantification. The experiment was performed on three biological replicates with their respective three technical replicates. Different letters indicate significant differences between samples according to Tukey's multiple comparison tests at *P* < 0.05. **(B)** Modulation of *pAtGRDP2::GFP-GUS* expression patterns by IAA. Seven day-old seedlings were treated with 0, 1, and 10 μM of IAA for 3 and 6 h. Images show representative individuals of two independent lines of *pAtGRDP2::GFP-GUS* (line 1 and 2). Images were taken on a Leica microscope at 10X magnification and recorded using the Leica Application Suite 3.0.0 software. **(C)** Expression analysis of auxin-related genes. qRT-PCR was performed in 15 day-old seedlings of Col-0, *Atgrdp2*-1, and -*OE3* overexpression line. Ten seedlings of each line per triplicate were used. For the qRT-PCR analysis it was used SYBR green dye, with the respective technical replicates. Normalized fold change was calculated comparing the target gene expression with a control (Col-0), after normalization to the Arabidopsis *UBQ5* gene using the (2^−ΔΔCt^) method. In case of ratios lower than 1, the inverse of the ratio was estimated and the sign was changed.

All previous experiments showed that the *Atgrdp2-1* and *amiR-1* lines have a delay in development; it might be possible that hormonal pathways controlling development are affected. Interestingly, Goda et al. (2004) reported that the *AtGRDP2* belongs to a group of genes specifically regulated by indole-3-acetic acid (IAA). We analyzed IAA levels in WT, mutant and overexpression lines (Figure 3A), and we observed that the *35S::AtGRDP2-OE2* and *-OE4* over-expression lines accumulated the highest IAA levels (Figure 3A). Furthermore, the effect of auxins on *AtGRDP2* gene expression was evaluated in the *pAtGRDP2::GFP-GUS-1* reporter line. GUS histochemical analyses were carried out in 7 day-old *pAtGRDP2::GFP-GUS-1* and *-2* transgenic lines treated with 0, 1, and 10 μM IAA for 3 and 6 h (Figure 3B). Plant treatments with IAA show increases in GUS signal in the root-hypocotyl junction (collet), and in the root differentiation zone in comparison to non-treated plants. In addition, IAA induces GUS signal in the root elongation zone; however, no signal is detected in root meristematic zone in control and treated seedlings (Figure 3B).

*AtGRDP2* appears to be an auxin-regulated gene, and the difference in IAA concentrations might be responsible for the accelerated growth rate phenotype observed in *AtGRDP2* overexpression lines. The relation between auxins and *AtGRDP2* gene was also explored by measuring the expression of Auxin Response Factors (*ARF2*, *ARF6, ARF8*), *miR167*, and the auxin transporter *AUX1* by qRT-PCR. Interestingly, the *ARF6*, *ARF8*, and *AUX1* genes are induced in the *35S::AtGRDP2-OE3* line and repressed in the *Atgrdp2-1* mutant (Figure 3C); opposite to *ARF2* which is induced in the *Atgrdp2-1* line. The *miR167*, which is a negative regulator of *ARF6* and *ARF8* transcription factors, was found up-regulated in the *Atgrdp2* mutant, and decreased in 35S::*AtGRDP2* overexpression line (Figure 3C).

### Overexpression of *AtGRDP2* in Arabidopsis improves recovery after salt stress

**Figure 4:**
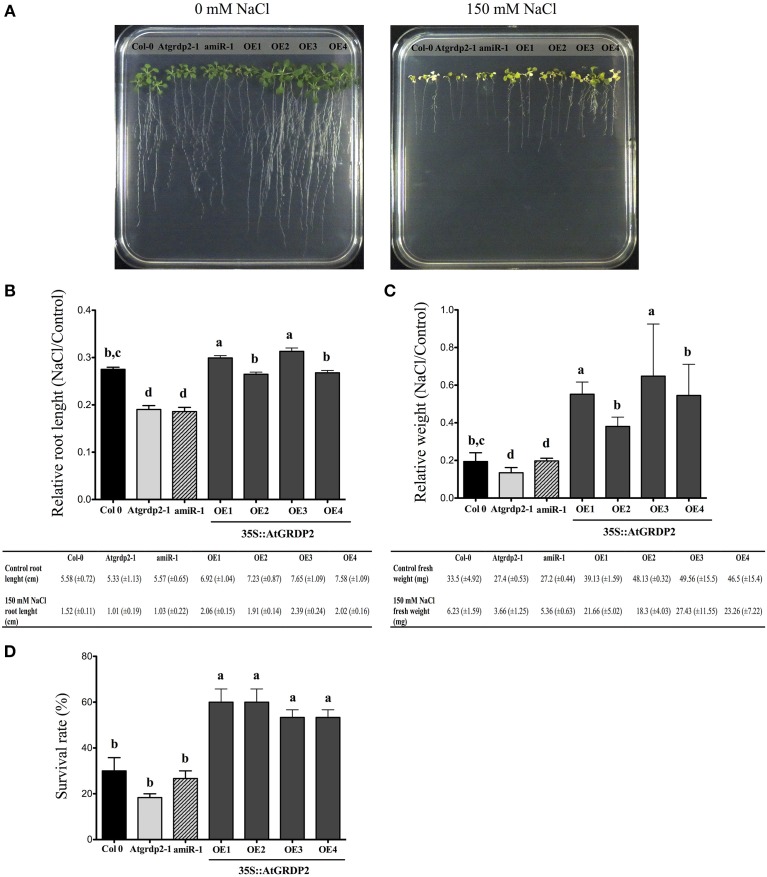
**Effect of salt stress on Arabidopsis Col-0, *Atgrdp2-1*, *amiR-1*, and *AtGRDP2* overexpression lines. (A)** Photographs of Arabidopsis (21 day-old) seedlings grown in 0.5x MS medium with 0 and 150 mM NaCl for 7 days **(B)** Data of primary root length, and relative root length (treated with 150 mM NaCl/untreated) for each line was represented graphically. **(C)** Data of fresh weight, and relative fresh weight (treated with 150 mM NaCl/untreated) for each line was represented graphically. **(D)** Survival rate of the Col-0, *Atgrdp2-1*, *amiR-1*, and AtGRDP2 overexpression lines after 7 days of salt stress recovery. Data are mean ± SE (*n* = 10) from three replicates. Different letters indicate significant differences (*P* < 0.05) among lines were explored through Tukey's multiple comparisons tests.

Two-week old Arabidopsis Col-0, *Atgrdp2* mutants and overexpression lines were grown 7 days in 150 mM NaCl (Figure 4A). An evident salt stress sensitivity phenotype was observed in the mutant lines (Figure 4A). The relative primary root growth was slower for *Atgrdp2-1* and *amiR-1* compared with WT. The relative fresh weight was higher for *35S::AtGRDP2-OE* overexpression lines (Figures 4B,D). After salt stress treatments, plantlets were transferred into sterile soil and the survival rate was calculated following a 7 days period of recovery. Plant survival rates indicate that more than 50% of the overexpression lines recovered while only 20% of *Atgrdp2*-*1* was able to survive (Figure 4C). Our data show clear salt stress tolerance in *AtGRDP2* overexpression lines.

**Figure 5:**
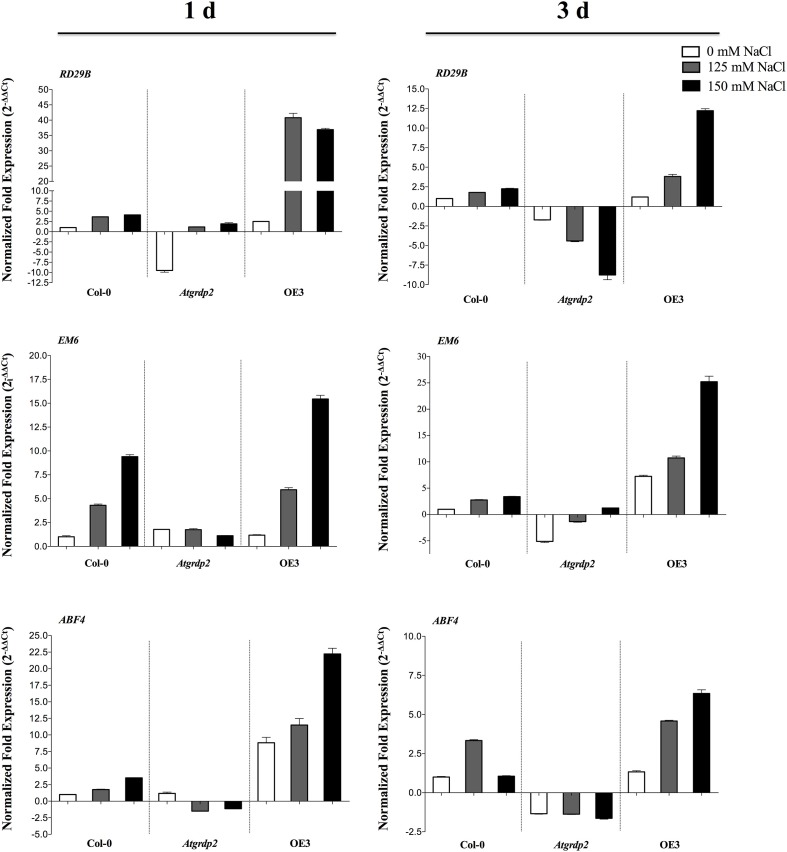
**Expression levels of *RD29B, EM6*, and *ABF4* genes in Col-0, *Atgrdp2-1*, and *35S::AtGRDP2-OE3* overexpression line during salt treatment**. Fourteen days old plants grown on 0.5X MS plates were transferred to 0.5X MS medium containing 0, 125, and 150 mM NaCl, for 1 and 3 days. Ten seedlings of each line per triplicate were used. The expression levels were estimated by qRT-PCR with the respective three technical replicates using SYBR green dye. Normalized fold change was calculated comparing the target gene expression (under salt stress) with a control (untreated Col-0), after normalization to the Arabidopsis *UBQ5* gene using the (2^−ΔΔCt^) method. In case of ratios lower than 1, the inverse of the ratio was estimated and the sign was changed.

Furthermore, genes known to be induced by abiotic stress were analyzed by qRT-PCR in 15 day-old Col-0, *Atgrdp2-1* mutant and *35S::AtGRDP2-OE3* overexpression lines grown 1 and 3 days under 125 and 150 mM NaCl. Expression levels of the selected genes: Responsive to Desiccation 29B (*RD29B*), Arabidopsis Early Methionine-Labeled 6 (*EM6/LEA*) and ABRE Binding Factor 4 (*ABF4*) are shown in Figure 5. These genes are ABA responsive and are mainly induced by stress conditions that involve cellular dehydration. In the *35S*::*AtGRDP2-OE3* overexpression line, *EM6*, *ABF4*, and *RD29B* genes were up-regulated under salt treatments at both times in comparison to WT plants. In contrast, all the analyzed genes were repressed in *Atgrdp2-1* (Figure 5).

### *AtGRDP2* overexpression in lettuce alters growth, development, and flowering time

**Figure 6:**
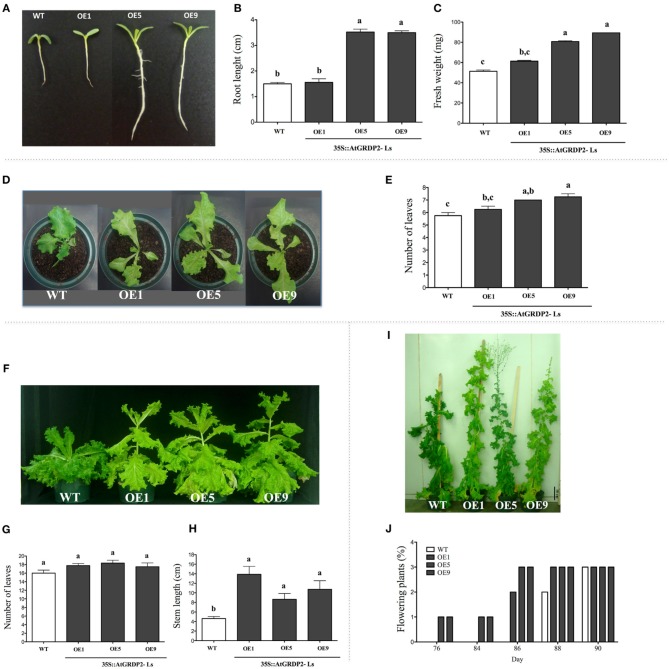
**Generation of lettuce transgenic plants overexpressing the Arabidopsis AtGRDP2 gene**. **(A)** Phenotype of 7 day-old WT and transgenic lettuce plants; **(B)** primary root length, and **(C)** fresh weight of WT and transgenic lettuce plants was measured. **(D)** 30 day-old WT and transgenic lettuce plants, **(E)** the number of leaves in these plants is graphically represented. **(F)** 60 day-old WT and transgenic lettuce plants; **(G)** the number of leaves and **(H)** stem length are indicated. **(I)** 90 day-old WT and transgenic lettuce plants; **(J)** distribution of flowering plants in a period of 15 days is graphically represented. Data was obtained from 9 seedlings of each line, mean ± SE (*n* = 9). Different letters indicate significant differences (*P* < 0.05) among lines were explored through Tukey's multiple comparisons tests.

Transgenic lettuce T3 homozygous lines that overexpress the *AtGRDP2* gene were generated, and *AtGRDP2* expression was estimated by qRT-PCR, observing different levels of *AtGRDP2* transcript in the lettuce *35S::AtGRDP2*-*Ls1*, -*Ls5*, and -*Ls9* lines. In WT lettuce, *AtGRDP2* expression was not detected as expected (Figure S3E). Overexpression of *AtGRDP2* gene positively affects the growth rate of lettuce plants at early stages of development (7 day-old plants; Figure 6A). The *35S::AtGRDP2-Ls5* and *-Ls9* exhibited faster growth, developed higher fresh weight and longer primary roots than WT plants (Figures 6A–C). In contrast, the *35S::AtGRDP2-Ls1* line, was phenotypically similar to the WT, with no statistical differences in fresh weight and primary root length (Figures 6A–C). The increased growth rate phenotype correlates with *AtGRDP2* gene expression in the lettuce transgenic lines (Figure 6A and Figure S3E). After, the transgenic and WT lettuce plantlets were grown in soil. As previously observed, 30 day-old plants of the *35S::AtGRDP2-Ls5* and *-Ls9* maintained the highest growth rates, and developed more leaves (Figure 6D). 60 day-old lettuce *AtGRDP2* overexpression lines (-*Ls1*, -*Ls5*, and *-Ls9*) were higher than WT lettuces, reflected in their stem length (Figures 6F–H). No differences were observed in the number of leaves among transgenic and WT at this stage of development (Figures 6F–H). Owing the accelerated growth rate of *AtGRDP2* transgenic lettuce lines, flowering time was also analyzed (Figures 6I,J). As expected, inflorescence development was accelerated in the transgenic lines. The *35S::AtGRDP2-Ls5* and *-Ls9* transgenic lettuce plants flowered 76 days after sowing, 12 days before the WT, while in the *35S::AtGRDP2-Ls1* plants the difference was minor, 2 days before WT flowering time (Figures 6I,J).

One interesting feature of the lettuce *35S::AtGRDP2* transgenic plants is the presence of light green leaves, in contrast to the dark green color observed in WT lettuce in all the developmental stages analyzed (Figure 6). For this reason, the chlorophyll content was estimated. No significant differences were observed in the total chlorophyll content between WT and transgenic plants (Figure S4). However, the chlorophyll *a/b* ratio was altered in transgenic plants, due to a higher chlorophyll *b* content (Figure S4). Furthermore, carotenoids content was measured. It was found that transgenic plants diminish their carotenoid content with age, i.e., 90 day-old-transgenic plants show up to 57% less carotenoids than WT lettuce plants (Figure S4C).

**Figure 7:**
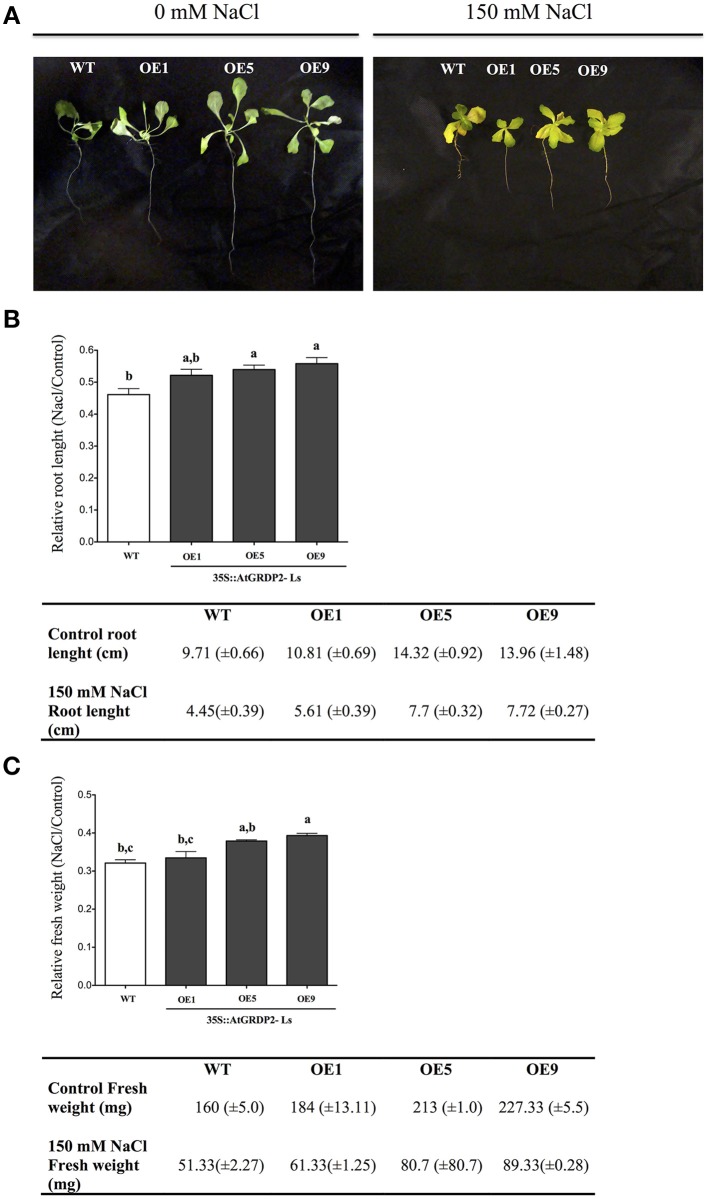
**Effect of salt stress on *AtGRDP2* lettuce transgenic plants**. WT and transgenic lettuce seeds were germinated in MS medium supplemented with 0 and 150 mM NaCl. **(A)** Photographs of lettuce plants after 21 days of salt stress. **(B)** Data of primary root length, and relative root length (treated whit 150 mM NaCl/untreated) for each line was represented graphically. **(C)** Data of fresh weight, and relative fresh weight (treated whit 150 mM NaCl/untreated) for each line was represented graphically. Data are obtained for 9 plants for each line, mean ± SE (*n* = 9). Different letters indicate significant differences (*P* < 0.05) among lines were explored through Tukey's multiple comparisons tests.

Finally, the response to salinity stress was evaluated in lettuce *AtGRDP2* overexpression lines (Figure 7) grown under 150 mM NaCl for 21 days. At this time, salt tolerance was noticed in the overexpression lines; which had longer roots and increased fresh weight in comparison to WT plants (Figures 7B,C).

## Discussion

In this study, we describe for the first time the *AtGRDP2* gene, which belongs to the DUF1399-GRDP family. *AtGRDP2* encodes a non-canonical glycine-rich protein of unknown function containing a DUF1399 domain, a putative RNA-binding motif and a glycine-rich domain. The three domains present in AtGRDP2 are conserved in other plant orthologous proteins (Figure S1). The Arabidopsis genome has three additional genes encoding proteins with a DUF1399 domain *AtGRDP1*, *At4g37682*, and *At1g56230* (Rodríguez-Hernández et al., 2014). *AtGRDP1* is involved in abiotic stress response and ABA signaling (Rodríguez-Hernández et al., 2014), while the latter are of unknown function. *AtGRDP2* and *AtGRDP1* seem to be paralogs (Rodríguez-Hernández et al., 2014), and show microsynteny (Kevei et al., 2005). The At4g37682 and At1g56230 proteins do not contain glycine-rich and RNP motifs, and share 46% and 15% identity respectively, with AtGRDP2 protein.

The canonical glycine rich proteins contain a high glycine percentage (from 40 to 70%), with arranged (Gly)_n_-X repetitions (Sachetto-Martins et al., 2000; Mousavi and Hotta, 2005). Besides the canonical GRPs, there are glycine-rich domain proteins (GRDPs), containing a short glycine-rich region. Several transcripts encoding GRDPs have been reported in *Eucalyptus* (Bocca et al., 2005), and recently our research group reported the *Phaseolus vulgaris PvGRDP1* gene, and the Arabidopsis *AtGRDP1* gene, which are induced under abiotic stress (Hernández-Lucero et al., 2014; Rodríguez-Hernández et al., 2014). The Arabidopsis *AtGRDP2* gene is developmentally regulated, with particularly high mRNA expression levels in buds, flowers, and immature siliques. In accordance, the Arabidopsis microarray database (Arabidopsis eFP Browser, www.bar.utoronto.ca) reports that *AtGRDP2* transcript is induced during stages 9, 10, 11, and 12 of flower development (Schmid et al., 2005). The expression of some canonical plant GRPs has been reported in floral organs. The oleosin-like protein GRP17 was identified as a component of the *A. thaliana* pollen coat required for rapid initiation of pollination (Mayfield and Preuss, 2000). Another characterized GRP, the AtOGB3 that has an oleosin domain, is required for pollen hydration (Mayfield and Preuss, 2000). Transcripts of Arabidopsis *GRPs* (*AtGRP1* and *AtGRP2*) were found to be abundant in flowers (de Oliveira et al., 1990). These studies suggest that proteins with glycine-rich regions play a role in flower organs development.

We found phenotypes of higher growth rates and development into vigorous plants in Arabidopsis and lettuce *AtGRDP2* overexpression lines, in contrast to the Arabidopsis *Atgrdp2-1*, which show an opposite behavior. The observed phenotypes are in accordance with different levels of *AtGRDP2* transcript accumulation, e.g., the *35S::AtGRDP2-OE3* line exhibited the longest primary roots, the highest fresh weight, and the highest transcript levels. In addition, flowering time was estimated under long- and short-day conditions, and it was found that Arabidopsis *AtGRDP2* overexpression lines flowered earlier than the parental plants. The *Atgrdp2-1* seedlings display a delayed flowering phenotype compared to WT plants. Our data show that *AtGRDP2* gene is involved in Arabidopsis growth and development. In accordance, the Arabidopsis AtGenExpress microarray database show *AtGRDP2* expression in shoot apex, inflorescence and apical meristems. Some reports related canonical GRPs to developmental processes. The vacuole-located glycine-rich protein AtGRP5 plays a role in organ growth possibly by promoting cell elongation processes (Mangeon et al., 2009). *AtGRP5* overexpression generated plants with longer roots and enhanced elongation of the inflorescence axis. Likewise, AtGRP7 (an RNA-binding protein) promotes floral transition in Arabidopsis through the autonomous pathway (Streitner et al., 2008).

Interestingly, the accelerated growth phenotype was noticed in both Arabidopsis and lettuce transgenic plants at different stages of development (seedling stage, vegetative, and reproductive growth). Deregulation of *AtGRDP2* gene levels affects growth and development, possibly due to hormonal changes. We found that Arabidopsis *AtGRDP2* overexpression lines show increased levels of the auxin indole-3-acetic acid (IAA). Unexpectedly, the *Atgrdp2-1* maintain similar levels of IAA as the WT. It might be that the other genes of the Arabidopsis DUF1399 family, mainly the AtGRDP1 paralog have functional redundancy with AtGRDP2. Auxins control several fundamental aspects of the plant development, such as cell division, cell expansion, pattern formation, root development, and apical dominance, and also environmental responses such as photo- and gravitropism (Berleth and Sachs, 2001; Woodward and Bartel, 2005). Microarray data published by Goda et al. (2004) revealed that the *AtGRDP2* belongs to a group of genes specifically regulated by IAA. Consistent with these observations, we found auxin response elements (ARF binding sites) in the *AtGRDP2* promoter (Figure S5), and IAA treatments of *AtGRDP2::GFP-GUS-1* seedlings show increased GUS expression in the root-hypocotyl junction and in the root differentiation zone.

Furthermore, we evaluated the expression of *ARF* transcription factors in Col-0, *Atgrdp2-1* and 35S::*AtGRDP2-OE3* overexpression lines. ARFs bind to auxin response promoter elements and mediate auxin dependent gene expression (Guilfoyle and Hagen, 2007). The lack and constitutive overexpression of *AtGRDP2* alters the expression of *ARF6* and *ARF8* regulators of floral development, showing an induction of both genes in 35S::*AtGRDP2-OE3* line, and a repression in the *Atgrdp2-1*. *ARF6* and *ARF8* are fined tuned by *miR167*, and the overexpression of *miR167* mimics *arf6/arf8* phenotypes of flowering delay (Wu et al., 2006; Rubio-Somoza and Weigel, 2013). *miR167* is induced in *Atgrdp2-1* and repressed in 35S::*AtGRDP2-OE3* lines. *miR167*, *ARF6*, and *ARF8* form part of a regulatory network that is essential for flower organ maturation and root development in Arabidopsis (Curaba et al., 2014). The expression patterns observed for *miR167*, *ARF6*, and *ARF8* might explain the phenotypes of delayed or accelerated flowering observed in the *Atgrdp2-1* mutant and overexpression lines. Another important auxin-regulated gene is *AUX1*, which encodes an auxin influx facilitator protein involved in polar auxin transport (Kramer and Bennett, 2006; Paponov et al., 2008). In the *35S::AtGRDP2-OE3* overexpression line, higher *AUX1* transcript levels are detected, suggesting a major auxin influx in these lines.

Recent studies refer a connection between auxin response and abiotic stress, by crosstalk with ABA signaling (Du et al., 2012, 2013). We observed that the *35S::AtGRDP2* overexpression lines with increased auxin levels are also more tolerant to salt stress. Conversely, *Atgrdp2*-*1* lines were susceptible to salt stress, and most mutant lines were unable to survive after stress treatments. In accordance, microarray data show that the *AtGRDP2* gene is induced by salt stress in Arabidopsis roots (Ma et al., 2006). Recently, we reported that the common bean *PvGRDP1* gene, orthologous to Arabidopsis *AtGRDP2* gene, was induced in leaves by salt stress at 2 and 5 days, but was down-regulated in stems after 2 days of treatment (Hernández-Lucero et al., 2014). The expression pattern of some stress marker genes was monitored between the *Atgrdp2-1* and *35S::AtGRDP2-OE3* overexpression lines under salt stress. We selected the transcription factor *ABF4* (ABRE Binding Factor 4), *Em6* (LEA-1), and *RD29B* (Responsive to Desiccation 29B) genes, which are induced by diverse abiotic stresses including salinity (Uno et al., 2000; Kang et al., 2002; Hundertmark and Hincha, 2008). Our results showed that salt treatment down-regulated most marker genes in the *Atgrdp2-1* line, in contrast to the increased expression noticed in the *35S::AtGRDP2-OE3* line at 1 and 3 days of salt stress. Shi et al. (2014) propose that auxin might contribute in the positive regulation of drought stress resistance, through regulation of root architecture, ABA-responsive genes expression, ROS metabolism, and metabolic homeostasis. They found that endogenous and exogenous auxin positively modulated the expression levels of multiple abiotic stress-related genes such as *RAB18*, *RD22*, *RD29A*, *RD29B*, *DREB2A*, and *DREB2B*. These data are in accordance with our observations in *AtGRDP2* overexpression lines that accumulate IAA, express higher levels of stress responsive genes, and become salt stress tolerant. As well, down regulation of *ARF2* in *35S::AtGRDP2-OE3* line might be related to stress tolerance. On the other hand, a slight induction of *ARF2* gene was observed in *Atgrdp2-1* background. *arf2* mutants have a delay in leaf senescence and are more resistant to oxidative stress (Lim et al., 2010).

It has been reported that *AtGRDP1* gene, paralog to *AtGRDP2*, is modulated to several abiotic stress, including salt treatment. The *Atgrdp1* mutant line was sensible to salt and osmotic stress during germination and cotyledon development, whereas *35S::AtGRDP1* over-expressing lines resulted in increased tolerance to abiotic stress. In addition, *35S::AtGRDP1* over-expressing lines were more resistant to ABA, resembling to well-known ABI phenotype, whereas disruption of the *AtGRDP1* gene resulted in ABA hypersensitivity (Rodríguez-Hernández et al., 2014).

*AtGRDP2* transgenic lettuce lines showed several interesting phenotypes, i.e., plants grow faster, tolerate salt stress, have lower content of carotenoids, and the chlorophyll *a/b* ratio is altered. Recently, Du et al. (2013) proposed that the balance of carotenoid, ABA and auxin homeostasis is critical for rice development and stress responses. Since lettuce is a high-value leafy vegetable grown commercially worldwide, transformed lines might have implications in the development of improved phenotypes given the high demand and the susceptibility of this crop to dehydration and salt stress (Martinez-Gonzalez et al., 2011; Kerbiriou et al., 2013; Kim et al., 2013).

It has been reported that canonical GRPs containing an RRM-GRPs can be modulated under abiotic stress (Kang et al., 2013; Yang et al., 2014). Such is the case of the Arabidopsis glycine-rich RNA-binding protein 2 (GRP2) that is involved in salt and cold stress tolerance during germination and seedling growth (Kim et al., 2007). Transgenic tobacco plants overexpressing the *Limonium bicolor* GRP gene are tolerant to salt stress, possibly due to increased superoxide dismutase and catalase activities and proline content (Wang et al., 2012). As well, overexpression of *AtGRP2* and *AtGRP7* in rice confers drought stress tolerance (Yang et al., 2014).

Herein, we present fast-growing Arabidopsis and lettuce transgenic plants that overexpress the *AtGRDP2* gene. AtGRDP2 belongs to a novel family of Arabidopsis proteins containing the DUF1399 domain. *AtGRDP2* gene is developmentally regulated showing particular expression patterns in Arabidopsis floral organs. It was found that *AtGRDP2* is an auxin-regulated gene. Deregulation of *AtGRDP2* in Arabidopsis mutant and overexpression lines affects the expression of key regulators of floral development (*ARF6*, *ARF8*, and *miR167*), leading to delay or acceleration of flowering in Arabidopsis. Increased auxin levels in *AtGRDP2* overexpression lines were correlated with salt stress tolerance and up-regulation of markers of stress response. Our data reveal an important role for *AtGRDP2* in plant growth and development, possibly through an auxin dependent mechanism.

### Conflict of interest statement

The authors declare that the research was conducted in the absence of any commercial or financial relationships that could be construed as a potential conflict of interest.

## Supplementary material

Figure S1 **The Arabidopsis Glycine Rich Domain Protein 2 (AtGRDP2). (A)** Schematic representation of AtGRDP2 domains: DUF1399, RNP-1, and GRD. The amino acid sequence of each domain is indicated within rectangles. **(B)** Unrooted phylogenetic tree of AtGRDP2 orthologous proteins and Arabidopsis proteins containing the DUF1399 domain (At2g22660, At1g56230, and At4g37682). The phylogenetic tree was created by the Neighbor Joining method of the PHYLIP 3.67 package (Felsenstein, 1989). Bootstrap support values out of 1000 pseudoreplicates of the data set and values are provided as percentages at the corresponding nodes when >50%. Phytozome accession numbers are as follows: Al, (*Arabidopsis lyrata*, 353047); Cr, (*Capsella rubella*, Carubv10004166m); Th, (*Thellungiella hallophila*, Thhalv10024448m); Br, (*Brassica rapa*, Bra010619); Cp (*Carica papaya*, 755.1); Ls (*Linum usitatissimum*, Lus10019239); Pt (*Populus trichocarpa*, POPTR_0015s04140.1); Me (*Manihot esculenta*, cassava4.1_002413m); Rc (*Ricinus communis*, 28333.m000558); Eg (*Eucalyptus grandis*, Eucgr.100602.1); Ppa (*Prunus persica*, ppa001356m); Mt (*Medicago truncatula*, Medtr5g030890.1); Pv, (*Phaseolus vulgaris*, Phvulv091027346m); Gm (*Glycine max*, Glyma01g02400); Cc (C*itrus clementina*, clementine0.9_002162m); Cs (*Citrus sinensis*, orange1.1g002907m); Ac (*Aquilegia coerulea*, Aquca_010_0041.1); Mg (*Mimulus guttatus*, mgv1a001553m); Os (*Oryza sativa*, Os11g40590); Bd (*Brachypodium distachyon*, Bradi4g13267); Si (*Setaria italica*, Si025845m); Sb (*Sorghum bicolor*, Sb05g025070); and Zm (*Zea mays*, GRMZM26358827_T01). **(C)** Schematic representation of the predicted *A. thaliana* DUF1399 proteins, AtGRDP1 (At2g22660), AtGRDP2 (At4g37900), At1g56230, and At4g37682. In the case of the AtGRDP1 and AtGDRP2 proteins, the RNP-1 motif and the glycine-rich domain (GRD) are indicated.

Figure S2 **Multiple sequence alignment of AtGRDP2 plant orthologous proteins**. Identical residues in the seven proteins are shown in black, and conserved substitutions are in gray. The conserved regions are underlined: the Domain of Unknown Function 1399 (DUF1399), the putative RNA binding motif -1 (RNP-1) and the glycine-rich domain (GRD). Pv (*P. vulgaris*, Phvulv091027346m); Gm (*G. max*, Glyma01g02400); Br (*B. rapa*, Bra010619); Th (*T. hallophila*, Thhalv10024448m); Cr (*C. rubella*, Carubv10004166m); Al (*A. lyrata*, 353047); At (*A. thaliana*, At4g37900).

Figure S3 **Gene structure and expression of AtGRDP2 gene in Col-0, *Atgrdp2-1*, *amiR-1* knockdown, and *35S::AtGRDP2* over-expression lines. (A)** Schematic representation of T-DNA insertion site (black triangle) at fourth exon in the Sail_387D04 mutant line. Exons are shown in black rectangles, and introns in solid lines. RT-PCR analysis of *AtGRDP2* gene in control and *Atgrdp2*-*1* mutant plants; Actin 8 gene was used as loading control. **(B)** Schematic representation of the pAmiR-AtGRDP2 vector. LB, left border for T-DNA integration; CaMV 35S, cauliflower mosaic virus 35S promoter; attB1 and attB2 sites for recombination; amiR-AtGRDP2, *A. thaliana* GRDP2 artificial microRNA; 3′-OCS, octopine synthase terminator; bar gene as herbicide resistance marker (BASTA); RB, right border for T-DNA integration. qRT-PCR expression levels of AtGRDP2 on three independent amiR-AtGRDP2 lines. **(C)** Schematic representation of the *35S::AtGRDP2* construct in pMDC32 binary vector. RB, right border for T-DNA integration; 2 × 35S, cauliflower mosaic virus 35S promoter; attB1 and attB2 sites for recombination; *AtGRDP2*, *A. thaliana* GRDP2 cDNA; nos T, nopaline synthase terminator region; HPTII, hygromycin resistance gene; LB, left border for T-DNA integration. **(D)** qRT-PCR expression levels of *AtGRDP2* in the Arabidopsis overexpression lines is represented as normalized fold change, and was calculated comparing the target gene expression with a control (Col-0), after normalization to the Arabidopsis *UBQ5* gene using the (2^−ΔΔCt^) method. **(E)** Relative gene expression levels of *AtGRDP2* gene in transgenic lettuce are presented as 2^−ΔCt^, where ΔCt = Ct_*AtGRDP2*_– Ct_*LsUBQ5*_. Bars represent mean ± SE (*n* = 3) of two experimental replicates.

Figure S4 **Chlorophyll and carotenoids content in WT and *AtGRDP2* transgenic lettuce lines**. Lettuce plants of 30 **(A)**, 60 **(B)** and, 90 day-old **(C)**, were analyzed for chlorophyll a and b, and carotenoids content. The relation of chlorophyll a/b was calculated. Data are mean ± SE (*n* = 4) with the respective three technical replicates. Letters indicate significant differences between samples according to Tukey's multiple comparison tests at *P* < 0.05.

Figure S5 **Putative auxin-responsive elements on AtGRDP2 promoter by using PLACE analysis**. Promoter of *AtGRDP2* (2000 bp) was analyzed in the PLACE database. In the schematic representation, ARFAT boxes (gray), and SURECOREATSULTR11 boxes (black), and its localization in bp are indicated.
