# Supplementary figures and images for: Overexpression of AtGRDP2, a novel glycine-rich domain protein, accelerates plant growth and improves stress tolerance
Source: Front Plant Sci. 2015 Jan 20;5:782. doi: 10.3389/fpls.2014.00782 (PMC4299439; doi:10.3389/fpls.2014.00782)

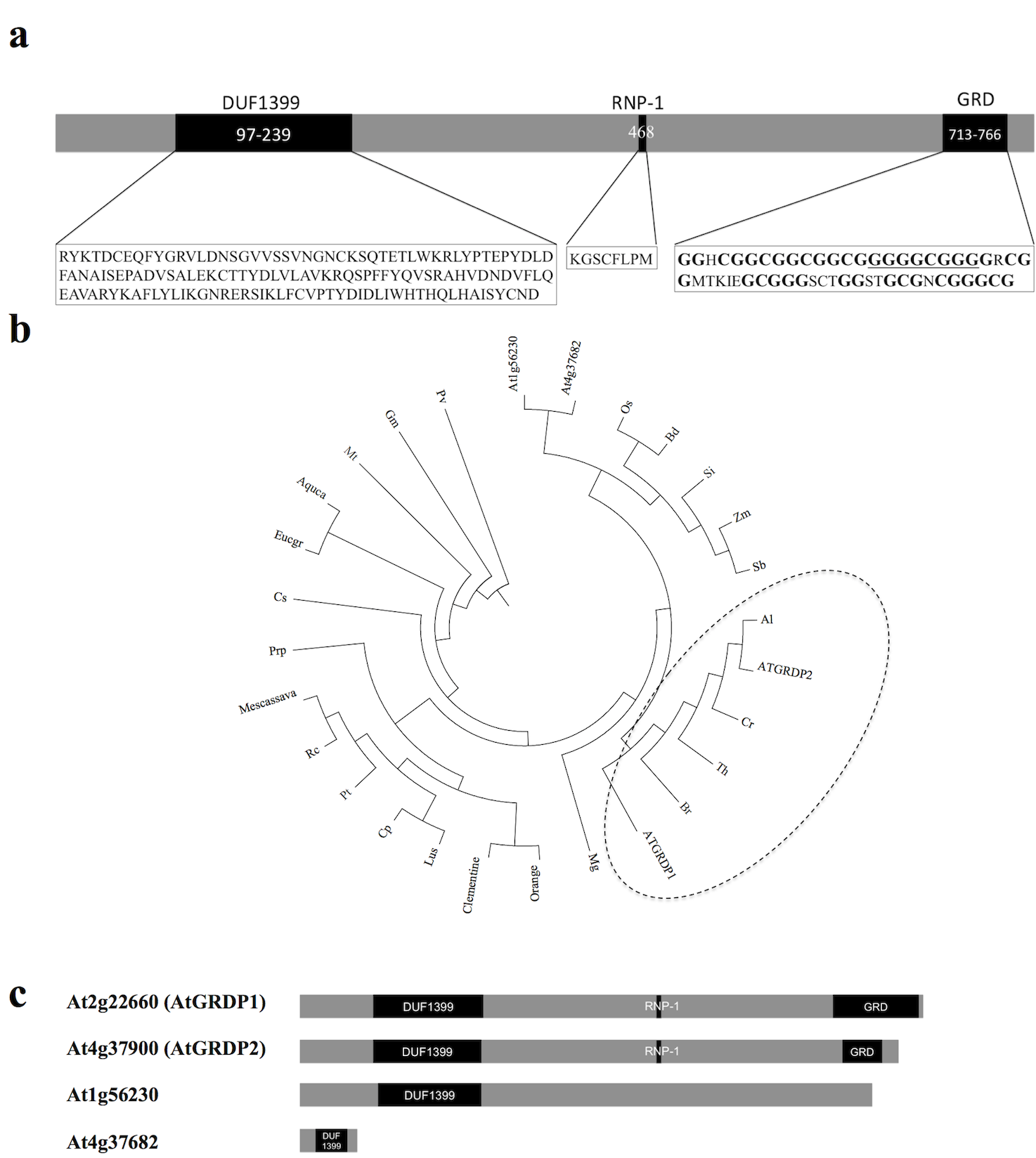

Supplement: Figure S1 — The Arabidopsis Glycine Rich Domain Protein 2 (AtGRDP2). (A) Schematic representation of AtGRDP2 domains: DUF1399, RNP-1, and GRD. The amino acid sequence of each domain is indicated within rectangles. (B) Unrooted phylogenetic tree of AtGRDP2 orthologous proteins and Arabidopsis proteins containing the DUF1399 domain (At2g22660, At1g56230, and At4g37682). The phylogenetic tree was created by the Neighbor Joining method of the PHYLIP 3.67 package (Felsenstein, 1989). Bootstrap support values out of 1000 pseudoreplicates of the data set and values are provided as percentages at the corresponding nodes when >50%. Phytozome accession numbers are as follows: Al, (Arabidopsis lyrata, 353047); Cr, (Capsella rubella, Carubv10004166m); Th, (Thellungiella hallophila, Thhalv10024448m); Br, (Brassica rapa, Bra010619); Cp (Carica papaya, 755.1); Ls (Linum usitatissimum, Lus10019239); Pt (Populus trichocarpa, POPTR_0015s04140.1); Me (Manihot esculenta, cassava4.1_002413m); Rc (Ricinus communis, 28333.m000558); Eg (Eucalyptus grandis, Eucgr.100602.1); Ppa (Prunus persica, ppa001356m); Mt (Medicago truncatula, Medtr5g030890.1); Pv, (Phaseolus vulgaris, Phvulv091027346m); Gm (Glycine max, Glyma01g02400); Cc (Citrus clementina, clementine0.9_002162m); Cs (Citrus sinensis, orange1.1g002907m); Ac (Aquilegia coerulea, Aquca_010_0041.1); Mg (Mimulus guttatus, mgv1a001553m); Os (Oryza sativa, Os11g40590); Bd (Brachypodium distachyon, Bradi4g13267); Si (Setaria italica, Si025845m); Sb (Sorghum bicolor, Sb05g025070); and Zm (Zea mays, GRMZM26358827_T01). (C) Schematic representation of the predicted A. thaliana DUF1399 proteins, AtGRDP1 (At2g22660), AtGRDP2 (At4g37900), At1g56230, and At4g37682. In the case of the AtGRDP1 and AtGDRP2 proteins, the RNP-1 motif and the glycine-rich domain (GRD) are indicated. [file Image1.TIFF]

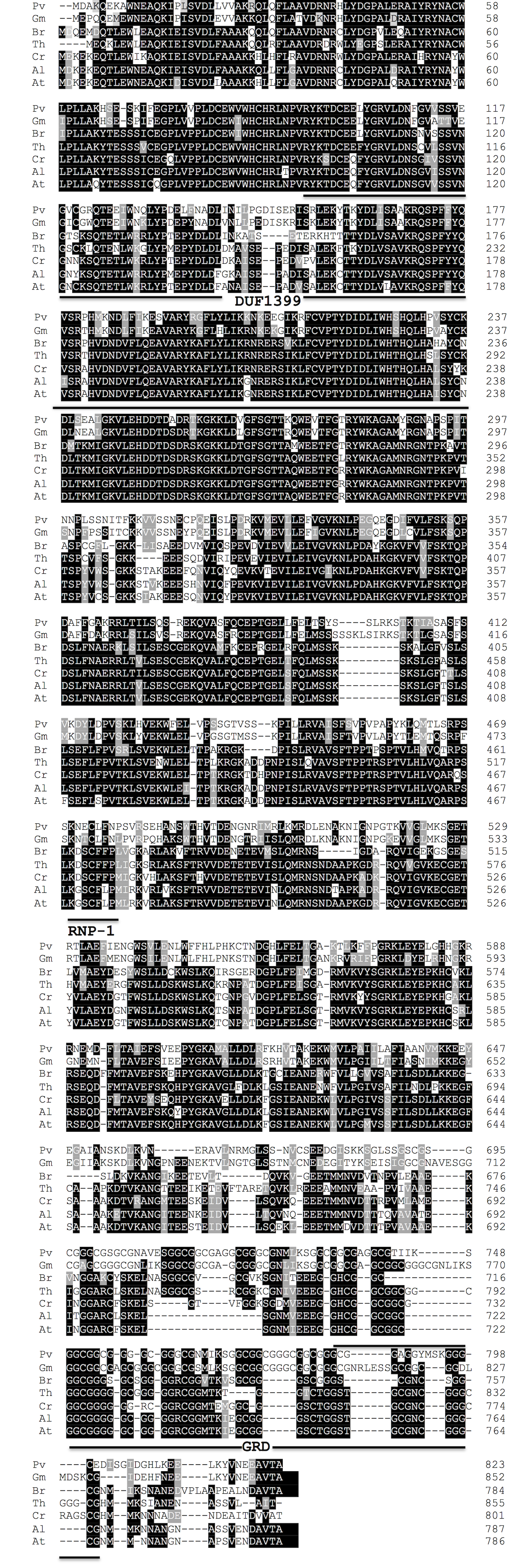

Supplement: Figure S2 — Multiple sequence alignment of AtGRDP2 plant orthologous proteins. Identical residues in the seven proteins are shown in black, and conserved substitutions are in gray. The conserved regions are underlined: the Domain of Unknown Function 1399 (DUF1399), the putative RNA binding motif -1 (RNP-1) and the glycine-rich domain (GRD). Pv (P. vulgaris, Phvulv091027346m); Gm (G. max, Glyma01g02400); Br (B. rapa, Bra010619); Th (T. hallophila, Thhalv10024448m); Cr (C. rubella, Carubv10004166m); Al (A. lyrata, 353047); At (A. thaliana, At4g37900). [file Image2.TIFF]

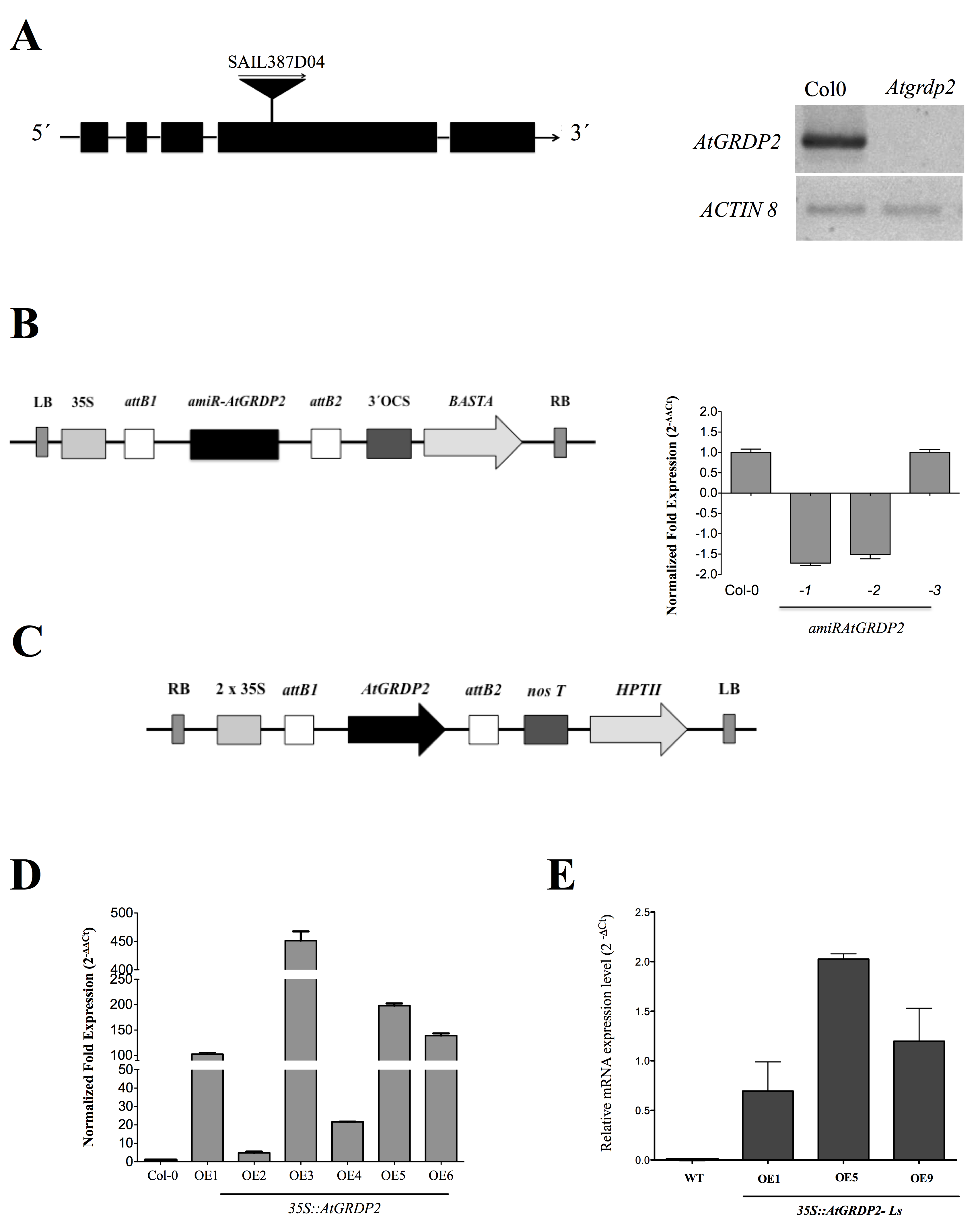

Supplement: Figure S3 — Gene structure and expression of AtGRDP2 gene in Col-0, Atgrdp2-1, amiR-1 knockdown, and 35S::AtGRDP2 over-expression lines. (A) Schematic representation of T-DNA insertion site (black triangle) at fourth exon in the Sail_387D04 mutant line. Exons are shown in black rectangles, and introns in solid lines. RT-PCR analysis of AtGRDP2 gene in control and Atgrdp2-1 mutant plants; Actin 8 gene was used as loading control. (B) Schematic representation of the pAmiR-AtGRDP2 vector. LB, left border for T-DNA integration; CaMV 35S, cauliflower mosaic virus 35S promoter; attB1 and attB2 sites for recombination; amiR-AtGRDP2, A. thaliana GRDP2 artificial microRNA; 3′-OCS, octopine synthase terminator; bar gene as herbicide resistance marker (BASTA); RB, right border for T-DNA integration. qRT-PCR expression levels of AtGRDP2 on three independent amiR-AtGRDP2 lines. (C) Schematic representation of the 35S::AtGRDP2 construct in pMDC32 binary vector. RB, right border for T-DNA integration; 2 × 35S, cauliflower mosaic virus 35S promoter; attB1 and attB2 sites for recombination; AtGRDP2, A. thaliana GRDP2 cDNA; nos T, nopaline synthase terminator region; HPTII, hygromycin resistance gene; LB, left border for T-DNA integration. (D) qRT-PCR expression levels of AtGRDP2 in the Arabidopsis overexpression lines is represented as normalized fold change, and was calculated comparing the target gene expression with a control (Col-0), after normalization to the Arabidopsis UBQ5 gene using the (2−ΔΔCt) method. (E) Relative gene expression levels of AtGRDP2 gene in transgenic lettuce are presented as 2−ΔCt, where ΔCt = CtAtGRDP2– CtLsUBQ5. Bars represent mean ± SE (n = 3) of two experimental replicates. [file Image3.TIFF]

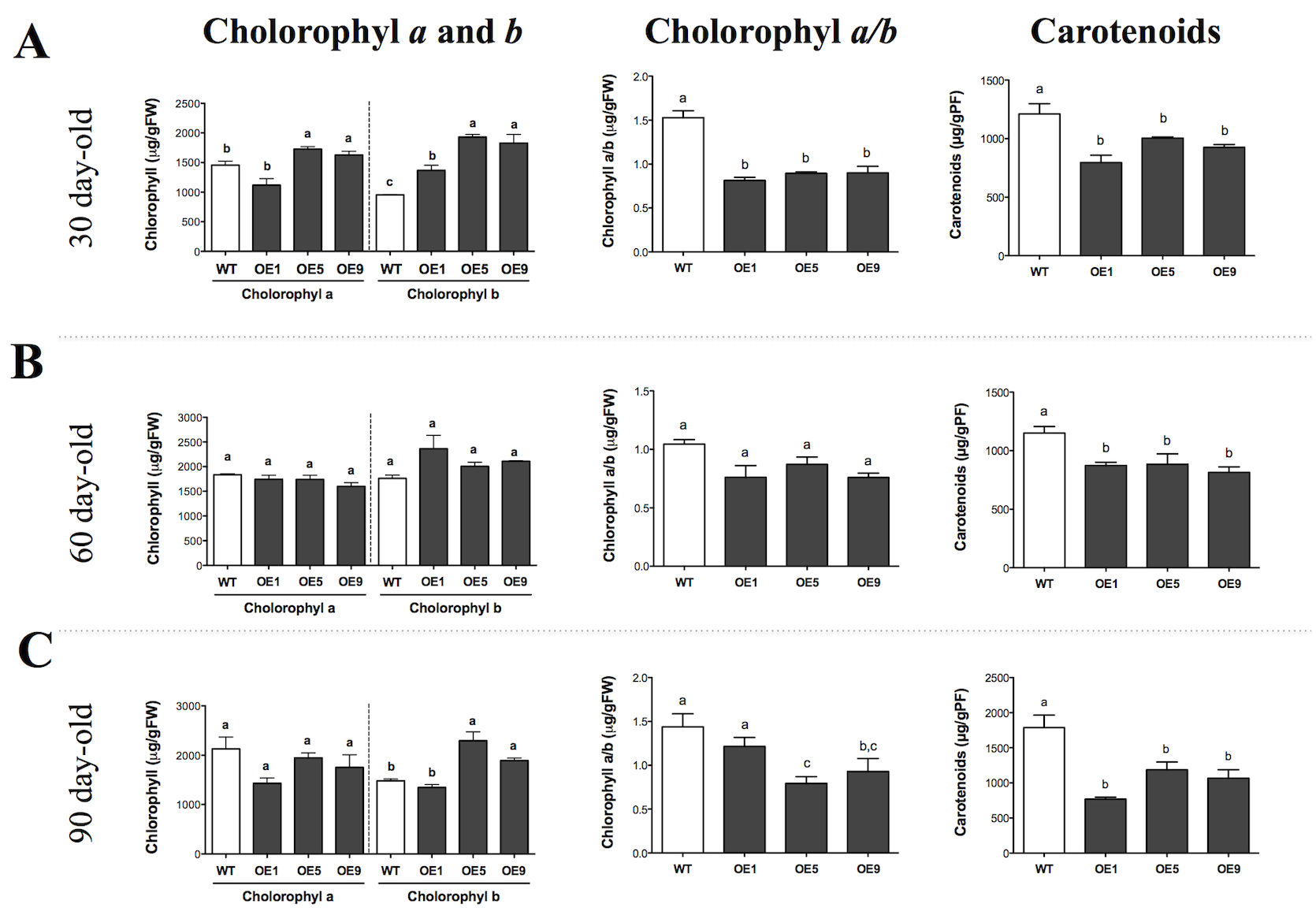

Supplement: Figure S4 — Chlorophyll and carotenoids content in WT and AtGRDP2 transgenic lettuce lines. Lettuce plants of 30 (A), 60 (B) and, 90 day-old (C), were analyzed for chlorophyll a and b, and carotenoids content. The relation of chlorophyll a/b was calculated. Data are mean ± SE (n = 4) with the respective three technical replicates. Letters indicate significant differences between samples according to Tukey's multiple comparison tests at P < 0.05. [file Image4.TIFF]

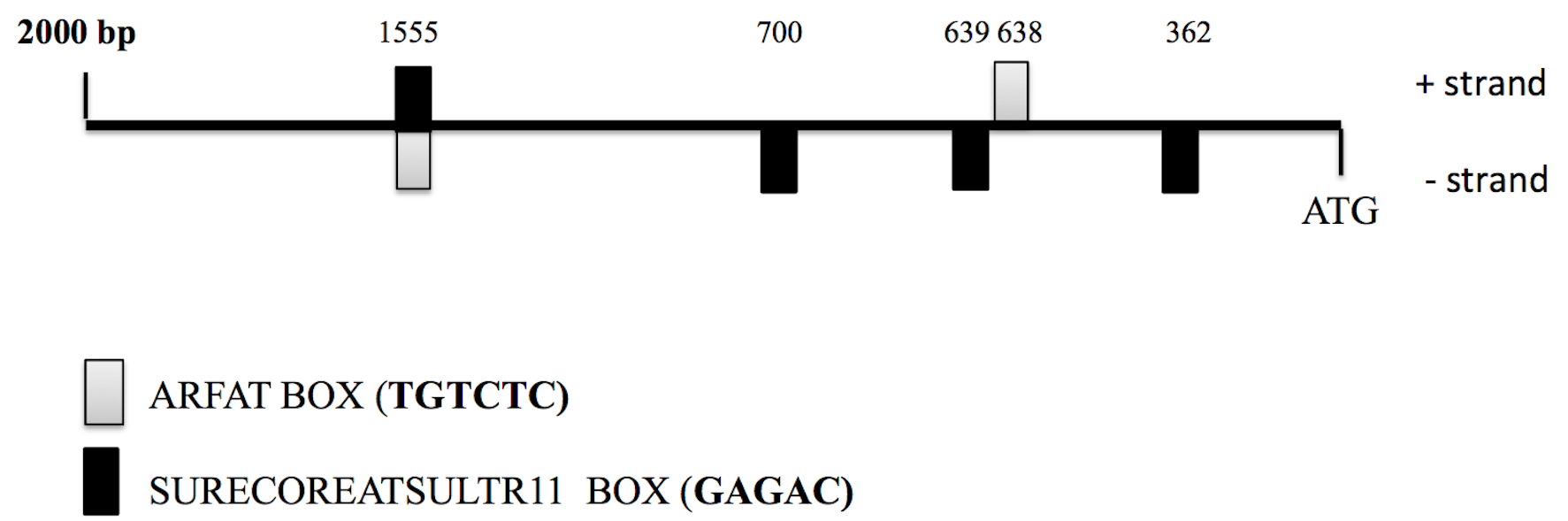

Supplement: Figure S5 — Putative auxin-responsive elements on AtGRDP2 promoter by using PLACE analysis. Promoter of AtGRDP2 (2000 bp) was analyzed in the PLACE database. In the schematic representation, ARFAT boxes (gray), and SURECOREATSULTR11 boxes (black), and its localization in bp are indicated. [file Image5.TIFF]
