# Supplementary material for: Overexpression of AtGRDP2, a novel glycine-rich domain protein, accelerates plant growth and improves stress tolerance
Source: Front Plant Sci. 2015 Jan 20;5:782. doi: 10.3389/fpls.2014.00782 (PMC4299439; doi:10.3389/fpls.2014.00782)
Supplement: Supplementary file 6 [file Table1.PDF]

Table S1. Primers used for cloning and expression analysis of the *AtGRDP2* gene

Application	Name	Sequence	Amplicon size (bp)
Amplification of *AtGRDP2* ORF	AtGRDP2-ORF-F	5'-GTAAGAGATGGACAAGGAAAAGGAG-3'	2377
	AtGRDP2-ORF-R	5'-CGACGCAGTTACAGCTTGATTATTC-3'	
T-DNA typification	Sail387D04-F	5'-CCGACTTACGATATTGATCTTATCTGGCAC-3'	960
	Sail387D04-R	5'-CCAGAAAGTGCCATCATACTCTGCC-3'	
Internal control for RT-PCR and qPCR analysis	ACT8-F	5'-GCCAGTGGTCGTACAACCG-3'	155
	ACT8-R	5'- TCATGAGGTAATCAGTAAGGTCAC-3'	
qPCR analysis of *AtGRDP2* transcript	AtGRDP2-F	5'-CCCAAAGCCAGTCACAACTTCTCC-3'	202
	AtGRDP2-R	5'- CAGAGAATCTGGTTGAGTTTTGCTGAAC-3'	
Internal control for RT-PCR and qPCR analysis	UBQ5-F	5'-TCGACGCTTCATCTCGTCCT-3'	155
	UBQ5-R	5'-CGCTGAACCTTTCCAGATCC-3'	
Internal control for RT-PCR and qPCR analysis	LsUBQ5-F	5'-TCAACCCTCCACCTGGTTCT-3'	155
	LsUBQ5-Rv	5'- CTCTGCACCTTTCCGGAATC -3'	
Amplification of *AtGRDP2* promoter	ATPROM37fw	5'-GGTCAAAGTTTGAAGGTTTG-3'	2022
	ATPROM37rv	5'-CTCTTACAAATTTTCACCTGCAA-3'	
qPCR analysis of *ARF2* transcript	ARF2-F	5'-CCGGGGACTTTAAGCTGTAG-3'	151
	ARF2-R	5'- GGCTTGCAGCAAAAGGGTTG-3'	
qPCR analysis of *ARF6* transcript	ARF6-F	5'-CTGTGACGACTTTGGGAACC-3'	163
	ARF6-R	5'-GTAATTAAACTAAGATGATCTCACTAC-3'	
qPCR analysis of *ARF8* transcript	ARF8-F	5'-TGTTTGCTATCGAAGGGTTGTTG-3'	135
	ARF8-R	5'- GTACCAAACGTTATTCACAAATGAC-3'	
qPCR analysis of *AUX1* transcript	AUX1-F	5'- CCACCGTCTTTGAGAGATCG -3'	164
	AUX1-R	5'- GAGCTTAGCACGCATTTAAAGG-3'	
qPCR analysis of *RD29B* transcript	RD29BFw	5'-ACAATCACTTGGCACCACCG-3'	170
	RD29BRv	5'-CAAAGTTCACAAACAGAGGCATC-3'	
qPCR analysis of *EM6* transcript	EM6Fw	5'-GGTACGGGAGGCAAAAGCTT-3'	117
	EM6Rv	5'- TTTGCGTCCCATCTGCTGATA -3'	
qPCR analysis of *ABF4* transcript	ABF4Fw	5'-ACCATGGTGAAGGATGAAGC-3'	174
	ABF4Rv	5'-AGGTCAGGGCACAAAATGTCA-3'	
qPCR analysis of miR167	miR167c	5'-TAAGCTGCCAGCATGATCTTG-3'	21
